# Loss of Sun2 promotes the progression of prostate cancer by regulating fatty acid oxidation

**DOI:** 10.18632/oncotarget.19210

**Published:** 2017-07-12

**Authors:** Cheng Yajun, Ye Chen, Li Xiaosa, Wang Xiao, Chen Jia, Wang Zhong, Xu Bin

**Affiliations:** ^1^ Department of Urology, Shanghai Ninth People's Hospital, Shanghai Jiaotong University School of Medicine, Shanghai, China; ^2^ Department of Urology, Changhai Hospital, Second Military Medical University, Shanghai, China; ^3^ School of Life Science and Technology, ShanghaiTech University, Shanghai, China

**Keywords:** prostate cancer, fatty acid oxidation, Sun2, SAA1, overall survival

## Abstract

The role of Sun2 has been described by previous studies in various types of cancers, including breast cancer and lung cancer. However, its role and potential molecular mechanism in the progression of prostate cancer have not been fully elucidated. In the present study, we found that Sun2 expression was reduced in prostate cancer tissues compared with paired normal tissues, and that low expression of Sun2 was significantly correlated with Higher Gleason scores, postoperative T stage (pT), Lymph nodal invasion and Clinical pathological stages. In addition, reduced Sun2 Expression predicts poor survival of prostate cancer patients and could serve as an independent predictor of prostate cancer patients overall survival (OS).Furthermore, Sun2 overexpression inhibits the prostate cancer cells growth, and Sun2 knockdown promotes the prostate cancer cells growth both *in vitro* and *vivo*. Mechanical silencing of , Sun2 promoted fatty acid oxidation (FAO) in prostate cancer, prostate cancer cells growth promoted by Sun2 silencing could be reversed by the FAO inhibitor Etomoxir. Additionally, we also showed that serum amyloid A1 (SAA1) play a vital role in FAO, ATP and cell growth promoted by Sun2 loss in prostate cancer. These results suggest that Loss of Sun2 promoted the prostate cancer progression by regulating FAO.

## INTRODUCTION

Cancer is the leading cause of death since 2010 and one of the major public health problem in china [1]. With advances in biopsy technology and increasing incidence of prostate-specific antigen screening or others factors, more cases of prostate cancer have been diagnosedin past decade [2]. Although traditional therapies, including surgical resection, endocrinotherapy and chemotherapyare still effective therapies for prostate cancer, transitions of prostate cancer to castration-resistant prostate cancer (CRPC) remains a lethal disease and kills thousands of men every day [3]. For men, it is most frequently diagnosed cancer and the second leading cause of cancer-death in western countries [4]. Serum prostate-specific antigen (PSA), as a Conventional prognostic factor, is already recognized as available biomarker for diagnosis and prognosis of prostate cancer. Numerousstudies have demonstrated that the prostate cancer patients with equivalent PSA level may have different clinical outcomes [5]. As prostate cancer is a heterogeneous multifocal disease with highly variable natural history, with respect to Age, race, geographic location, familial history and genetic background, [6], it is difficult to predict its initiation, progression and prognosis of prostate cancer, and it need us to find new biomarkers of prostate cancer to diagnose and predict clinical outcome [9–11]. Therefore, it need to identify specific biomarkers for diagnosis and novel target for therapy to improve prognosis and treatment of this cancer.

Nuclear envelope (NE) separates chromosomes from the cytoplasm in eukaryotic cells and NE proteins play a vital role in various biological effects by mediating genome organization, gene expression and mutatation [7]. The NE is a double lipid bilayer, comprising outer (ONM) and inner nuclear membranes (INM) that form the perinuclear space (PNS) [8]. The LINC (linker of nucleoskeleton and cytoskeleton) complex connects the nucleus with the cytoskeleton by assembling NE proteins. It consists of two kinds of protein families: nesprin protein and SUN protein, which was located in the outer and inner nuclear membrane respectively, interacting each other in PNS [9]. Additionally, SUN protein, consisting of Sun1 and Sun2, also interacts with lamins, functioning as nuclear skeleton in the nucleoplasm. Dysregulation of Sun2, as a characteristic NE protein, is associated with many human diseases, including cancers. Overexpression of Sun2 could prevent HIV infection by interfering with viral replication [10]. Recent studies have shown that SUN2 inhibit a variety of tumor malignancies *in vitro* and *in vivo*. Heish et al [11] reported that Sun2 was down-expressed in the central nervous system (CNS) embryonal tumors asa novel tumor suppressor. And miR-221/222 promoted the central nervous system embryonal tumorigenesis bytargeting Sun2. Sun2 expression was also decreased in breast cancer and may play a tumor suppressor role [12]. Furthermore, Xiao-bin et al [13] demonstrated that downregulation of Sun2 promoted lung cancer progression by regulating the Warburg effect. However, expression and role of SUN2 in prostate cancer is unkown and needs to be explored.

To discover new therapeutic targets for prostate cancer, it is essential for exploring the molecular mechanisms underlyingtumor progression. The aim of the present study was to examine the relationship between Sun2 expression and clinicopathologic outcomes by tissue microarray (TMA) of 90 prostate cancer patients, and then investigate the role of Sun2 in prostate cancer cell growth *in vitro* and *in vivo*. In addition, the impact of Sun2 on the metabolism of prostate cancer cells was also evaluated. Finally, RNA sequence was used to explore the potential mechanism of prostate cancer progression inhibited by Sun2.

## RESULTS

### Sun2 is downregulated in prostate cancer and correlates with clinicopathological parameters

**Figure 1:**
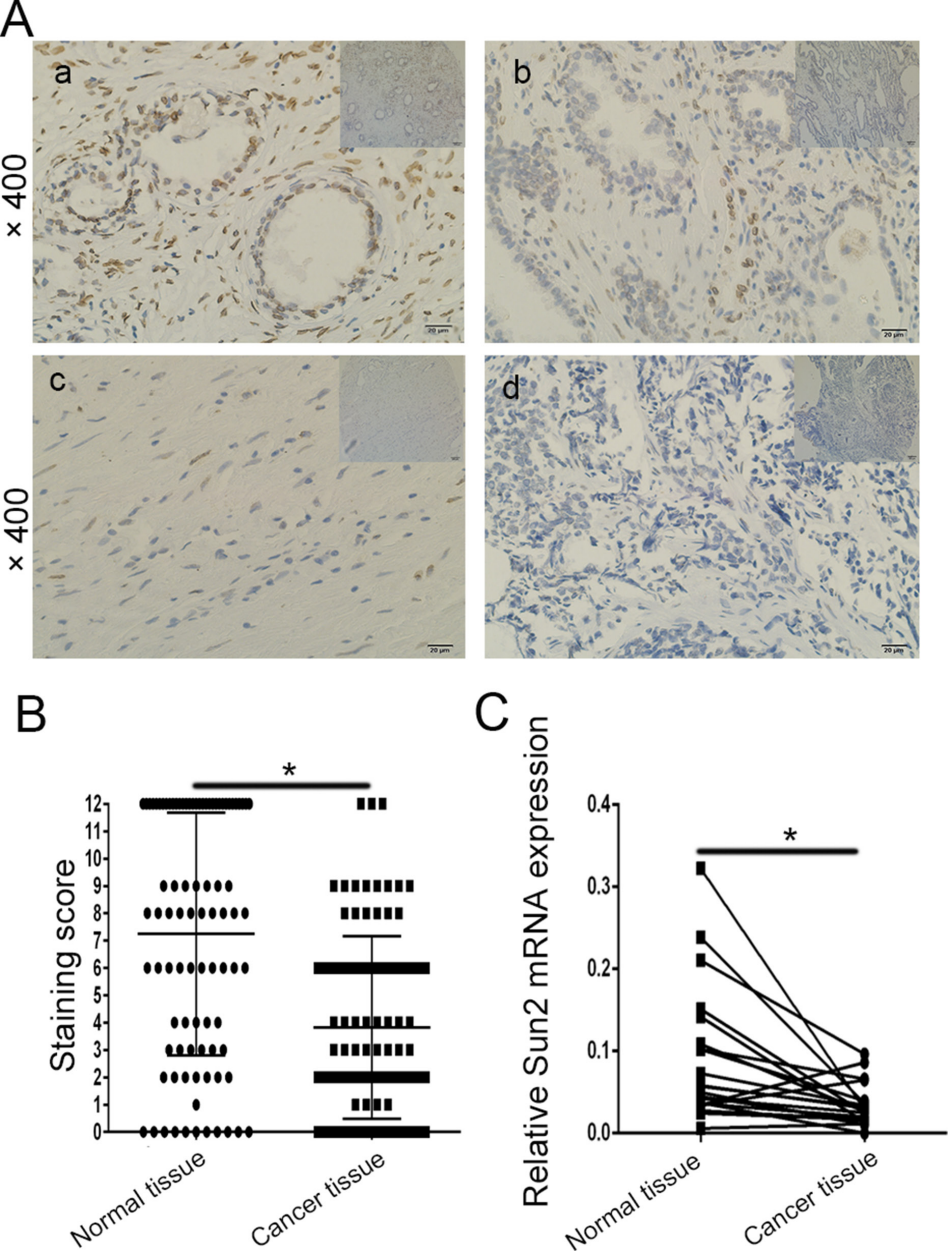
The expression pattern of Sun2 in prostate cancer tissues (**A**) a: Normal tissues (positive), b-d: immunohistochemistry staining of Sun2 in prostate cancer samples, b (positive), c (medium), d (weak); (**B**) The mean staining score of TTP in prostate cancer tumor tissues *vs.*matched adjacent normal tissues (*n* = 90); (**C**) The mean expression of TTP mRNA in prostate cancer tissues *vs.*matched adjacent normal tissues (*n* = 35). *P < 0.05, **P < 0.01.

To examine whether the Sun2 protein was differentially expressed in prostate cancer tissues *vs.* normal tissues, immunohistochemical staining and qRT-PCR were performed. As shown in Figure 1A, Sun2 protein was mainly localized in the nucleus. The results of staining scoring in the 90 prostate cancer tissues and adjacent benign tissues showed that Sun2 expression was downregulated in prostate cancer (61/90,67.5%) (7.24 ± 4.42 *vs.* 3 .86 ± 3.3, *P* < 0.05) (Figure 1B). Furthermore, Sun2 mRNA expression was also downregulated (26/35, 74.3%) in prostate cancer patients (*P* < 0.05) (Figure 1C). We further analyzed the relationship between the expression levels of Sun2 and several clinicopathological features of prostate cancer patients, and found that low expression of Sun2 was significantly correlated with Gleason score (*P* = 0.018), Postoperative T stage (pT) (*P* = 0.03), Lymph nodal invasion (*P* = 0.004) and clinical pathological stage (*P* = 0.012) (Table 1). No significant association was seen between age, PSA at diagnosis and Sun2 expression (Table 1).

**Table 1:** Correlation between the clinicopathologic variables and Sun2 expression in prostate caner

Clinical character	Cases	Sun2 expression	Low	*χ*^2^value	*p* value
		High			
**Age (years)**					
< 60	31 (34.4%)	11 (35.5%)	20 (64.5%)	0.23	0.64
≥ 60	59 (65.6%)	18 (30.5%)	41 (69.5%)		
**Gleason score**					
< 8	58 (64.5%)	24 (41.3%)	34 (58.7%)	6.26	0.018*
≥ 8	32 (35.5%)	5 (15.7%)	27 (84.3%)		
**PSA at diagnosis (ng/ml)**					
< 10	41 (45.6%)	10 (24.4%)	31 (75.6%)	2.11	0.178
≥ 10	49 (54.4%)	19 (38.8%)	30 (61.2%)		
**Tumor infiltration**					
T1–T2	50 (55.6%)	20 (33.3%)	30 (66.7%)	5.31	0.03*
T3–T4	40 (44.4%)	9 (66.2%)	31 (33.8%)		
**Lymph nodal status**					
Negative	48 (53.3%)	22 (45.8%)	26 (54.2%)	8.7	0.004**
Positive	42 (46.7%)	7 (16.7%)	35 (83.3%)		
**Clinical pathological stage**					
< T2C	54 (60%)	23 (44.6%)	31 (55.4%)	6.65	0.012*
≥ T2C	36 (40%)	6 (16.7%)	30 (83.3%)		
**Total**	90 (100.0%)	29 (32.2%)	61 (67.8%)		

Statistical analyses were carried out using Pearson χ^2^ test.

*P < 0.05 and **P < 0.01 were considered significant.

### Decreased Sun2 expression predicts poor survival in prostate cancer and is an independent predictor of OS

**Figure 2:**
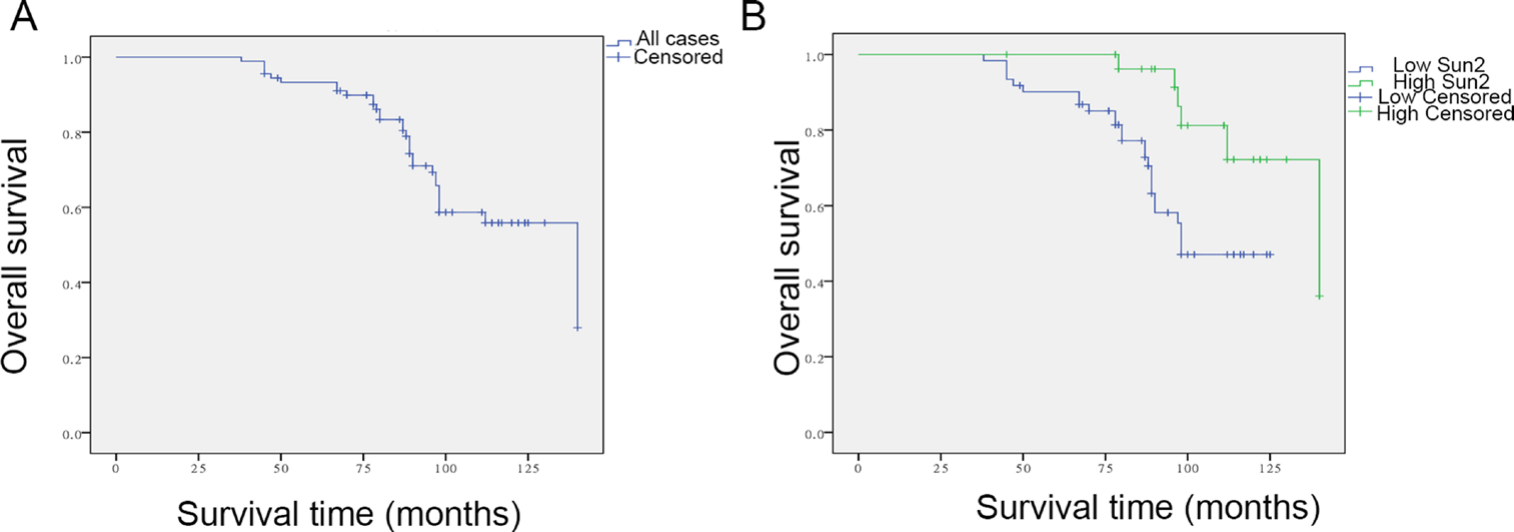
Relationship between low Sun2 expression and outcome of patients with prostate cancer (**A**) Kaplan-Meier curves for overall survival (OS) of 90 prostate cancer patients. (**B**) Kaplan-Meier curves for OS in 90 prostate cancer patients with low or no expression or high expression of Sun2. *P < 0.05, **P < 0.01.

To assess the relationship between Sun2 expression and the prognosis of prostate cancer patient, Kaplan-Meier survival analysis was performed and the results showed that low Sun2 expression predicted poor survival of prostate cancer patients (*P* < 0.01) (Figure 2). Meanwhile, follow-up time was no significantly correlated with high or low Sun2 expression (m ± sd, 85.4 ± 23.6 *vs.* 89.6 ± 32.4, *P* > 0.05). Moreover, univariate analysis showed that Sun2 expression (*P* < 0.01), Gleason score (*P* = 0.02), Lymph nodal invasion(*P* < 0.01) and TNM Stage (*P* < 0.01) were the factors affecting the survival rate in postoperative prostate cancer patients (Table 2). Next, multivariate analysis was performed and the results showed that low Sun2 expression (*P* < 0.01) and TNM stage (*P* = 0.02) were the independent factors predicting the prognosis of prostate cancer patients.

**Table 2:** Univariate and multivariate analysis of overall survival of prostate cancer patients

Clinical character	Univariate analysis			Multivariate analysis		
	HR	(95% CI)	*p* value	HR	(95% CI)	*p* value
**Sun2 expression**	3.84	2.1–6.98	< 0.01**	3.01	1.61–5.89	< 0.01**
**Age (years)**	1.12	0.8–1.54	0.80			
**Gleason score**	2.34	1.44–3.56	0.02*	1.45	0.79–2.97	0.28
**PSA at diagnosis**	1.43	0.87–1.98	0.69			
**Tumor infiltration**	1.86	0.97–2.04	0.06			
**Lymph Nodal status**	2.57	1.51–3.61	< 0.01**	1.67	0.87–3.04	0.49
**TNM staging**	3.25	1.97–5.15	< 0.01**	2.83	1.42–4.34	0.02*

### Sun2 overexpression inhibits the prostate cancer cell growth

**Figure 3:**
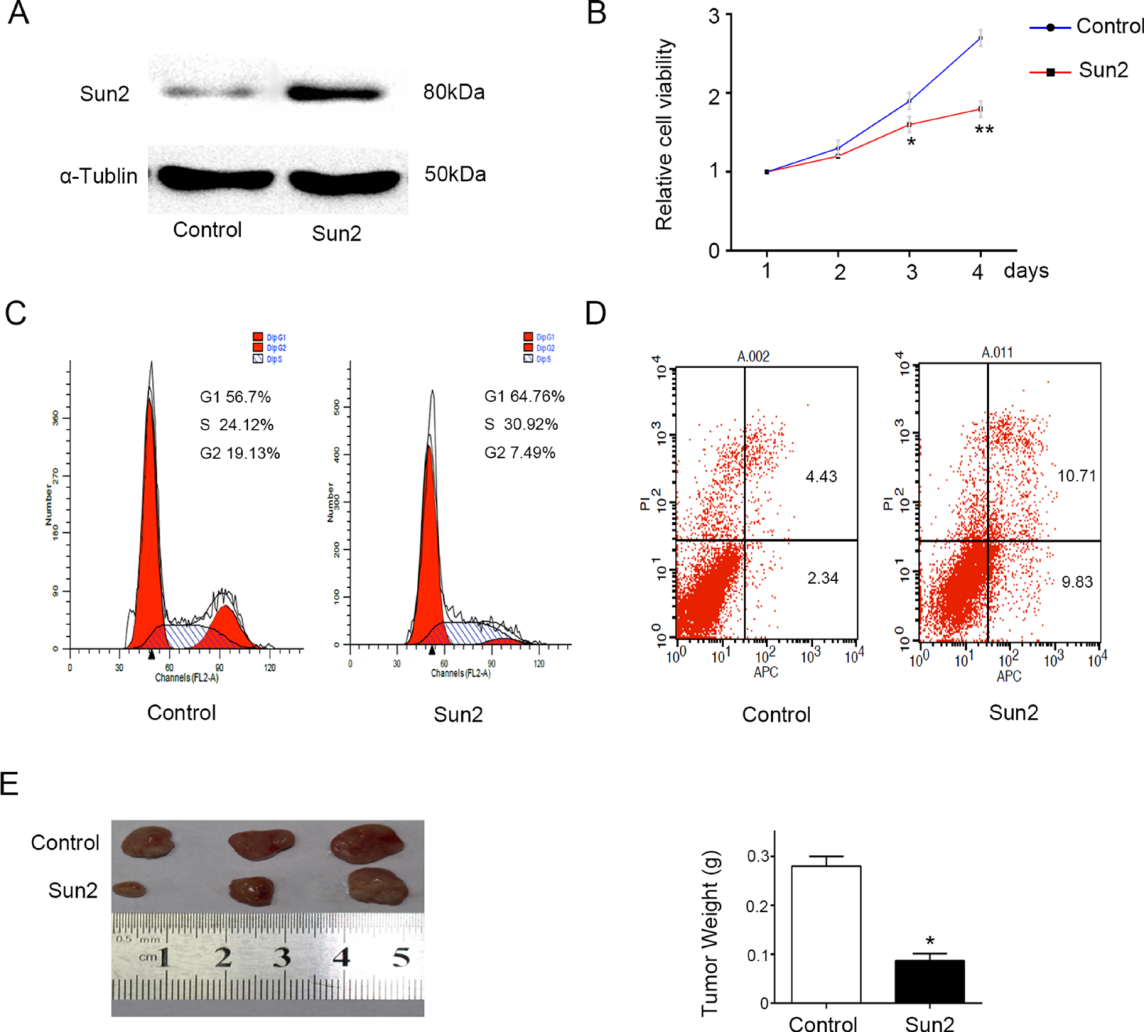
Sun2 overexpression inhibits prostate cancer growth (**A**) Western blot was used to confirm the Sun2 transfection efficiency. (**B**) Cell proliferation levels were measured using the cell counting kit-8 at 1, 2, 3 and 4 days. (**C**) and (**D**) Cell-cycle distribution and cell apoptosis were analyzed by flow cytometry, respectively. (**E**) Tumorigenicity assay in nude mice was performed in Sun2 overexpression stable cell lines *vs.* control. *P < 0.05, **P < 0.01.

In order to determine the role of Sun2 in prostate cancer, CRPC cell line pc-3 with relatively low expression Sun2 was transfected with Sun2. As shown in Figure 3A, Sun2 was overexpressed in PC-3 cells by western blot identification, and a stable line was established. Furthermore, we found that Sun2 overexpression inhibited the cancer cell proliferation by CCK-8 evaluation at 48 and 72 h (*P* < 0.05) (Figure 3B). Moreover, cell apoptosis and the cell cycle induced by Sun2 overexpression were assessed by flow cytometry. The results showed that Sun2 overexpression promoted the cancer cell apoptosis (*P* < 0.05) (Figure 3C) and induced cell cycle arrest in G1/S phase (*P* < 0.05) (Figure 3D) compared with the control. Finally, a xenograft tumor model was conducted to evaluate the growth of prostate cancer induced by Sun2. It was found that the mean tumor weight was significantly reduced in Sun2 overexpression tumors compared with vector control tumors (*p* < 0.05) (Figure 3E). The above results suggest that Sun2 inhibited the progression of prostate cancer.

### Sun2 silencing promotes the prostate cancer cell growth

**Figure 4:**
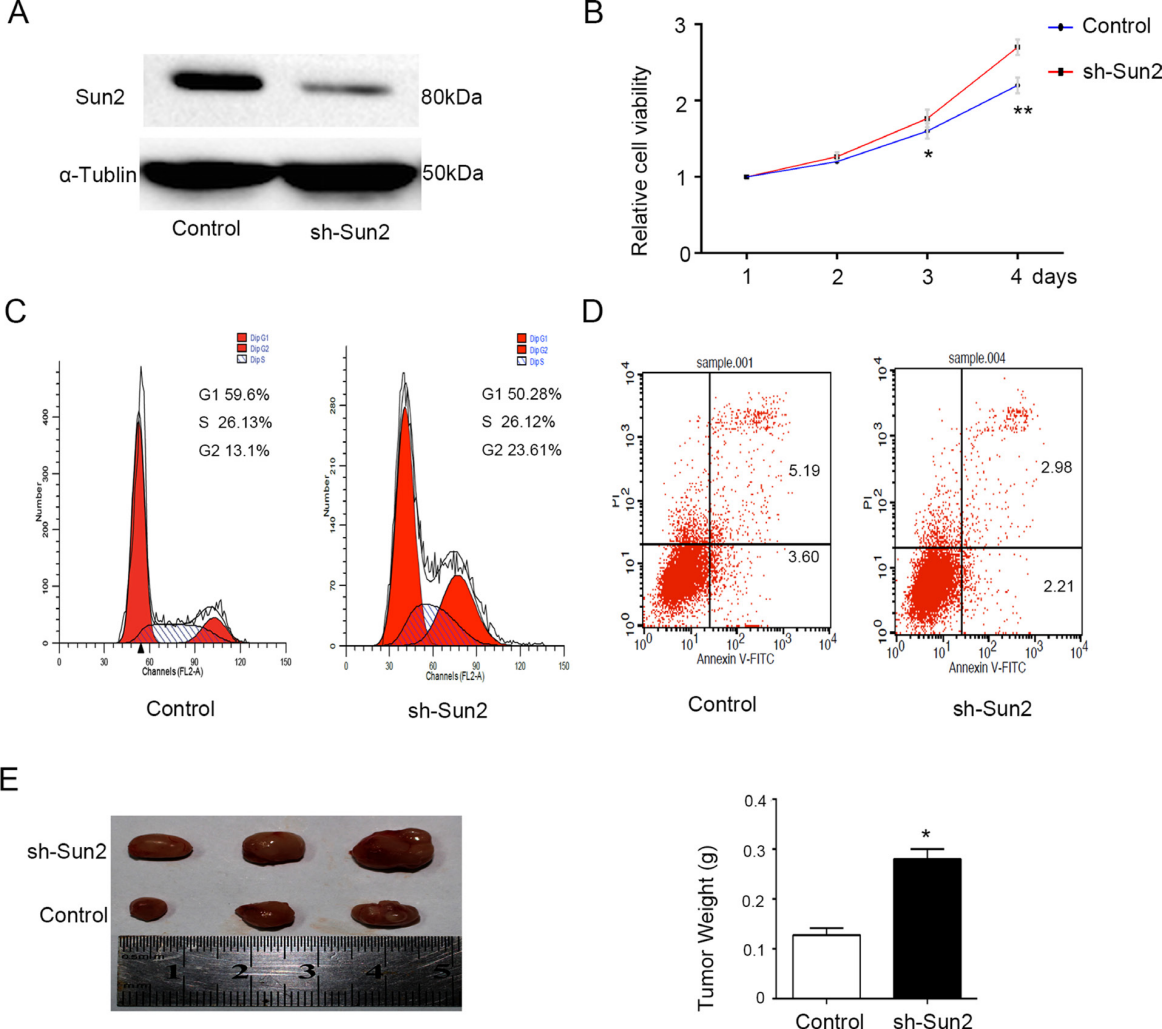
Sun2 knockdownpromotes the prostate cancer growth (**A**) Western blot was used to confirm the sh-Sun2 transfection efficiency. (**B**) Cell proliferation levels were measured using the cell counting kit-8 at 1, 2, 3 and 4 days. (**C**) and (**D**) Cell-cycle distribution and cell apoptosis were analyzed by flow cytometry, respectively. (**E**) Tumorigenicity assay in nude mice was performed in Sh-Sun2 downexpression stable cell lines *vs.*control. *P < 0.05, **P < 0.01.

CRPC cell line C4-2 with relatively high Sun2 expression was transfected with sh-Sun2. Sun2 protein expression after shRNA infection was determined by immunoblotting analysis (Figure 4A), showing that it was significantly decreased compared with control. The effect of Sun2 knockdown on rostate cancer cells proliferation was evaluated using CCK-8 kit. Cpt1c significantly inhibited cell growth in Sun2-shRNA transfected cells compared with the control at 48 and 72 h (*P* < 0.05) (Figure 4B). Moreover, we found that Sun2 silencing inhibited the cell apoptosis by flow cytometry analysis. Additionally, the number of cells in G1 and S phase (*P* < 0.05) was decreased and the number of cells in G2 phase was increased as compared with control (both *P* < 0.05) (Figure 4C, 4D). Finally, tumorigenicity assay in nude mice showed that the mean tumor weight was significantly increased in sh-Sun2 tumors as compared with the control (*p* < 0.05) (Figure 4E). These results suggest that Sun2 silencing promoted the progression of prostate cancer.

### Sun2 suppresses the fatty acid oxidation (FAO) in prostate cancer

**Figure 5:**
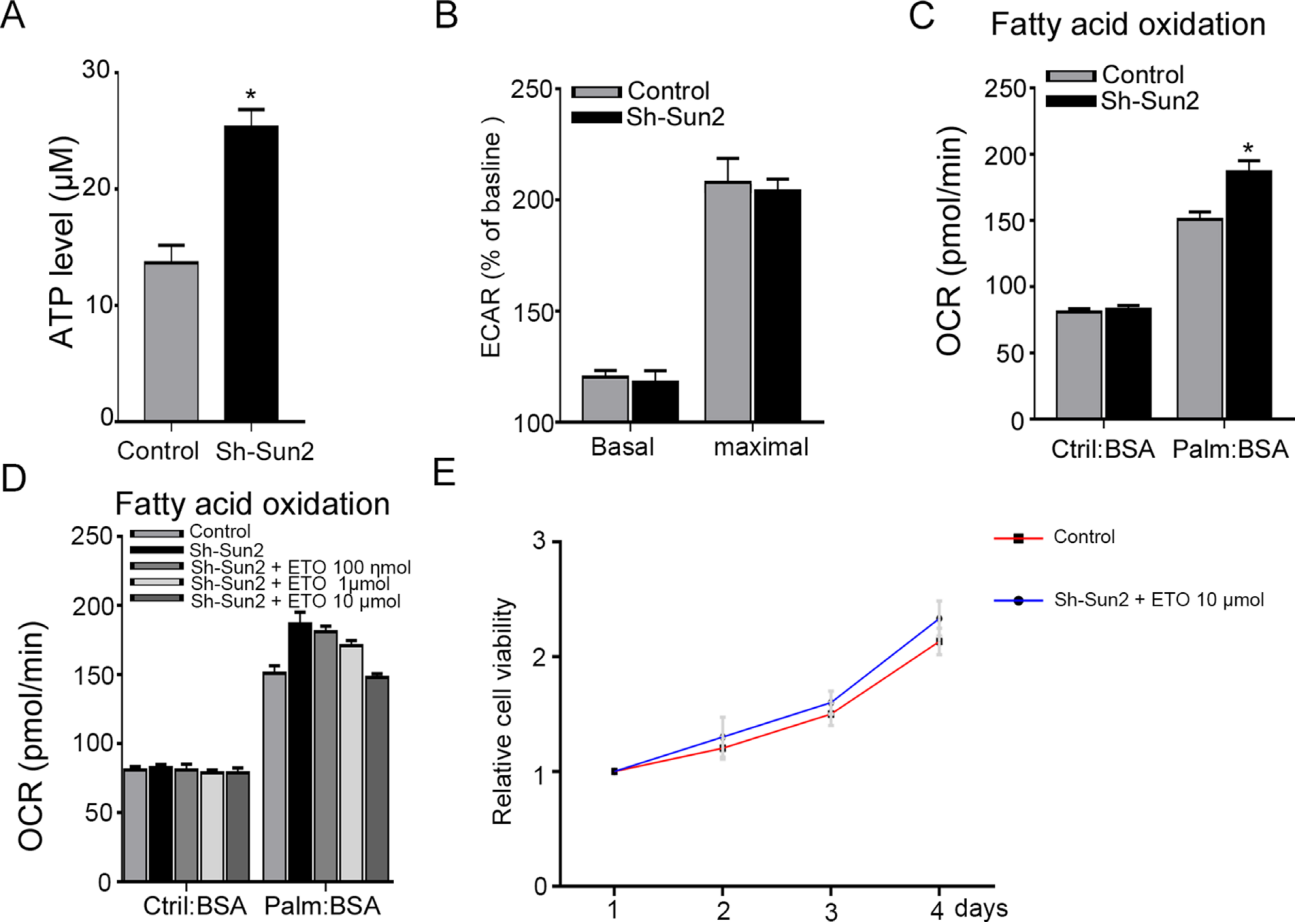
Sun2 suppresses the fatty acid oxidation (FAO) in prostate cancer (**A**) ATP concentrations were measured in sh-Sun2 prostate cancer cell lines and control. (**B**) The glycolytic rate was measured in sh-Sun2 prostate cancer cell lines and control. (**C**) FAO was measured in sh-Sun2 prostate cancer cell lines and control. (**D**) sh-Sun2 prostate cancer cell lines were cultures with ETO, 100 nmol, 1umol and 10 umol for 24 h, and FAO was measured. (**E**) sh-Sun2 prostate cancer cell lines were cultured with ETO10 umol, and cell proliferation was measured by CCK assay.*P < 0.05, **P < 0.01.

Sun2 can decrease the energy production by suppressing aerobic glycolysis of cancer cell as previously described [11], therefore, ATP concentrations was evaluated. Consistent with previous report, silencing Sun2 promoted the ATP production of prostate cancer cells (Figure 5A). Next, glucose uptake and the glycolytic rate was assessed to determine whether Sun2 had any impact on aerobic glycolysis. Our results showed that silencing Sun2 can not increase glucose uptake (data not shown) and the glycolytic rate (Figure 5B) in prostate cancer cells. Recent studies suggest that it relied on lipid oxidation more than on aerobic glycolysis to fulfill the energetic demands in prostate cancer [12]. Therefore, FAO was measured by Cellular OCR. We found that silencing Sun2 facilitated the FAO as compared with the control (Figure 5C). And FAO enhanced by low Sun2 expression could be downregulated by a FAO inhibiter ETO in a concentration- dependent manner (Figure 5D). Due to FAO facilitated by Sun2 silencingcould be neutralized by ETO, with 10μmol concentration, it was selected to culture with sh-Sun2 cell lines. The Results showed that growth of prostate cancer cells promoted by low Sun2 expression can be reversed by ETO at 10 μmol concentration (Figure 5E). These results suggest that low Sun2 promoted the prostate cancer progression by enhancing FAO rather aerobic glycolysis.

### The potential mechanism of FAO regulated by Sun2 in prostate camcer

**Figure 6:**
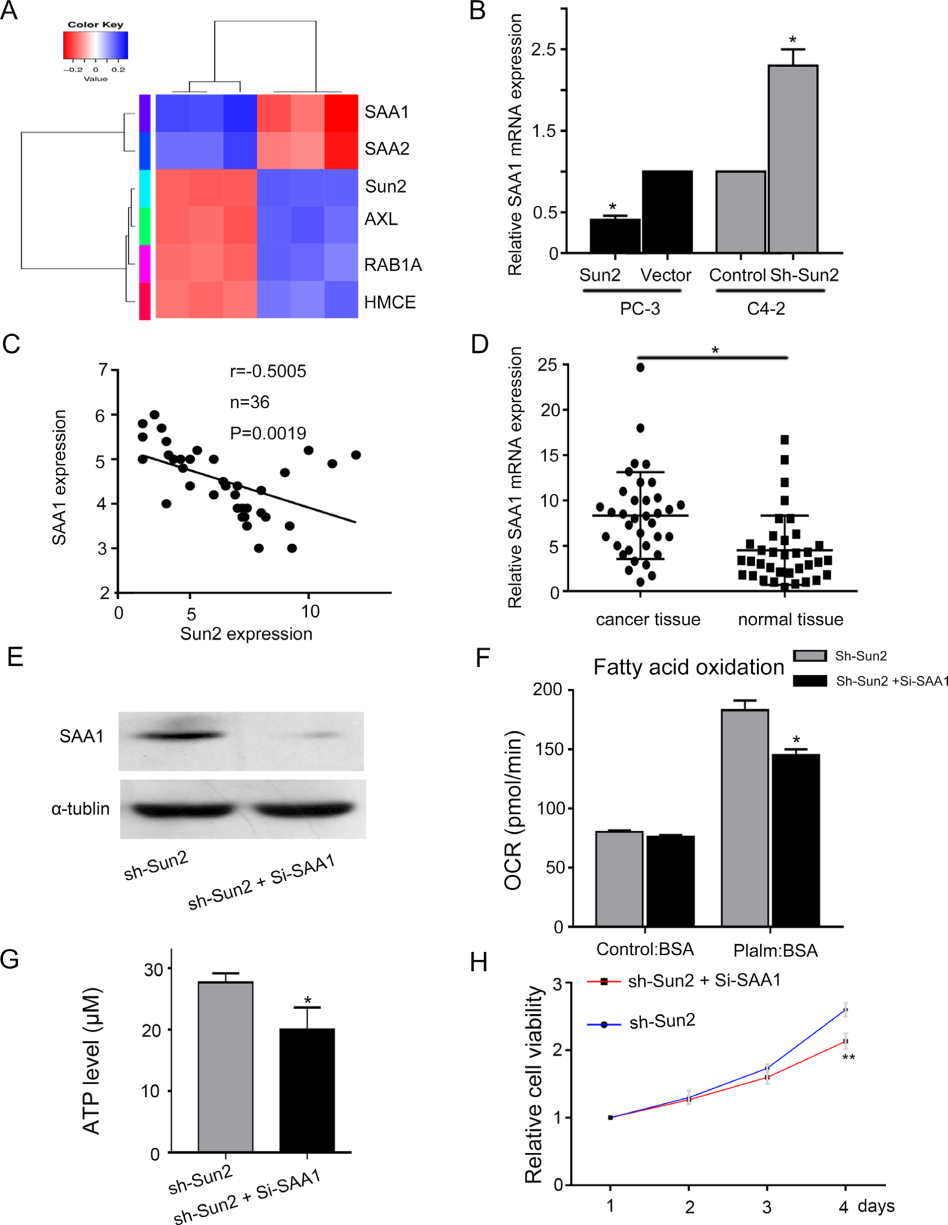
The underlying mechanism of FAO regulated by Sun 2 in prostate cancer (**A**) Differentially expression genes of RNA in sh-Sun2 and control areshown in the form of a heat map. (**B**) SAA1 expression was evaluated in overexpression and knockdown of Sun2 cell lines compared with control by RT-PCR analysis. (**C**) Correlation between Sun2 expression and SAA1 expression was observed in the prostate cancer tissues (*n* = 35). (**D**) Expression of SAA1 mRNA in prostate cancer tissues *vs., matched adjacent normal tissues* (*n=35*). (**E**) Si-SAA transfection efficiency was identified by western blot. (**F**) FAO was evaluated in sh-Sun2 stable cell lines with Si-SAA transfection *vs.* Sun2 stable cell lines. (**G**) ATP level was evaluated in sh-Sun2 stable cell lines with Si-SAA transfection *vs.* Sun2 stable cell lines. (**H**) Cell viability was evaluated in sh-Sun2 stable cell lines with Si-SAA transfection *vs.*Sun2 stable cell lines. *P < 0.05, **P < 0.01.

RNA Sequence analysis of sh-Sun2 and control prostate cancer cells was performed to explore mechanism of FAO regulated by Sun2 in prostate cancer. Serum amyloid A1 (SAA1), serum amyloid A2 (SAA2), receptor tyrosine kinase (AXL), Ras-related protein Rab-1A (RAB1A) and HMCES were identified (Figure 6A). Recent reports showed that SAA played an important role in lipid metabolism [14]. And we found that overexpression or knockdown of Sun2 decreased or increased SAA1 expression, respectively, in prostate cancer cells (Figure 6B). Next, we observed a significant inverse correlation between Sun2 and SAA1 in prostate cancer tissues (*P* = 0.0019) (Figure 6C). In addition, SAA1 was highly overexpressed in prostate cancer tissues as compared with normal tissues (*P* < 0.05) (Figure 6D). These results suggested that SAA1 may playan import role in prostate cancer progression regulated by Sun2. sh-Sun2 stable cell lines were transfected with Si-SAA1 and the transfection efficiency was determined by immunoblotting analysis, showing SAA1 expression was significantly decreased in sh-Sun2 stable cell lines were transfected with Si-SAA1 as compared with sh-Sun2 stable cell lines (Figure 6E). FAO assay showed that Si-SAA1 partially suppressed the FAO in sh-Sun2 state cell lines (*P* < 0.05) (Figure 6F). In addition, ATP level in sh-Sun2 stable cell lines could be decreased by Si-SAA1 downregulation (*P* < 0.05) (Figure 6G), and cell proliferation also be inhibited by Si-SAA1 downregulation (*P* < 0.05) (Figure 6H). These results suggest that FAO, ATP level and cell growth promoted by Sun2 loss was partially suppressed by SAA1.

## DISCUSSION

Recent studies [11–13] reported that Sun2 expression was downregulated in various cancers and also played a pivotal role in cellular biological pathways. The aim of the present study was to elucidate the expression and role of Sun2 in prostate cancer. We identified Sun2 as a gene that is down-expressed in prostate cancer tissues *vs.*normal tissues, and that low expression of Sun2 was significantly correlated with the high Gleason score, pT, Lymph nodal invasionand clinical pathological stage. Additionally, decreased Sun2 expression predicts poor survival in prostate cancer and wasan Independent predictor of OS. Next, Sun2 overexpression inhibited the prostate cancer growth, whileSun2 downregulation promoted the prostate cancer growth. These results point to an important role of Sun2 in prostate cancer progression, though the underlying molecular mechanism is unknown.

It is widely acknowledged that metabolic characteristics of elevated glucose uptake and lactate production occur in majority of tumor, is called the Warburg effect [15]. Recent reports revealed that aberrant expression or activation of gene promoted or inhibited the cancer initiation and progression by altering cancer cellular metabolism [16]. Sun2 plays a tumor suppressor role by inhibiting the Warburg effect in lung cancer as previously described. Here, we showed that low Sun2 expression promoted prostate cancer progression by enhancing FAObut had no significant impact on glycolysis. Prostate cancer, as a hormone-dependent cancer, grows slowly and is mainly dependent on lipid oxidation for fuel more than on aerobic glycolysis [17]. This is probably the reason why positron emission tomography (PET) with radiotracer18 F-fluorodeoxyglucose does not work well in prostate cancer and has been limited in imaging of prostate cancer diagnosis and follow-up [18]. Though the mechanisms by which prostate cancer cells use lipids to their benefit are poorly understood, now, this still represents a novel avenue to investigate new nontoxic therapeutic approaches to prostate cancer treatment. Schlaepfer et al. [19] reported that Etomoxir, as a clinic drug for heart failure treatment, decreased prostate cancer growth by blocking lipid oxidation. Lloyd et al. [20] showed that peroxisomal enzyme, α-methylacyl-CoA racemase (AMACR), which enhances the transformation of branched chain fatty acids to a form suitable for β-oxidation, were highly overexpressed in prostate cancer compared with normal prostate. Additionally, Lipid synthesis is increased in prostate cancer and Lipogenic enzyme, such as fatty acid synthase (FASN), ATP citrate lyase (ACLY) and acetyl-CoA carboxylase α (ACACA) is highly overexpressed in prostate cancer [21]. However, the mechanism of FAO regulated by Sun2 in prostate cancer is unclear.

To explore the mechanism of FAO regulated by Sun2 in prostate cancer, six genes (SAA1, SAA2, AXL, RAB1A and HMCE) were identified by RNA sequence. We observed significant a significant inverse correlation between Sun2 and SAA1 in prostate cancer. And FAO, ATP level and cell growth increasing in sh-Sun2-downexpressing prostate cancer cells could be reversed by SAA1 interference Serum amyloid A protein (SAA) is an apolipoprotein that can replace apolipoprotein A1 (apoA1) as the major apolipoprotein of high density lipoprotein (HDL). It is well recognized that SAA plays an important role in lipid metabolism, but how SAA impacts lipid metabolism remains incompletely understood. Cha H et al [22] reported that SAA increased the expression of CPT1 and reduced PPARγ expression to increase FA oxidation. These results suggested SAA1 plays an important role of FAO regulated by Sun2 in prostate cancer. However, how SAA1 expression regulated Sun2 in PCa is unknown, and we will explore the potential mechanism in the next step.

In summary, Sun2 expression is reduced in prostate cancer, and may prove to be a useful predictor of poor clinical outcomes of prostate cancer patients. Additionally, loss of Sun2 promotes the prostate cancer progression by enhancing FAO.

## MATERIALS AND METHODS

### Ethics statement

This experiment was approved by the institutional Ethical Committee of Shanghai Ninth Peoples’ Hospital. All patients signed informed consent provided by our team before initiation of the experiment.

### Patient’ specimens

Prostate sample including adjacent non-neoplastic tissues and primary tumors were obtained from 90 patients with prostate cancer, whounderwent radical prostatectomy in Shanghai Ninth peoples’ Hospital as the primary therapy (without hormonal or radiotherapy) between 2005 and 2010. 2010 pTNM AJCC classification and Gleason grading system were used to evaluate the tumors. In addition, no other relative treatment such as endocrinotherapy or radiotherapy were applied before surgery.

### Immunostaining and evaluation

Immunohistochemical staining was performed with the primary antibody for Sun2 (ab124916, Abcam, UK). The procedure details for immunohistochemical staining and immunostaining was described in our previous study [22]. A low-level Sun2 expression was defined when the staining score in cancer tissues was less than or equal to that in adjacent normal tissues. And high level was defined when staining score in cancerous tissues displayed a higher than that in adjacent normal tissue.

### Cell culture

Hormone-refractory prostate cancer cell lines, C4-2 and PC-3, were cultured in RPMI-1640 medium containing 10% FBS (Gibco, USA) and 100 U/ml penicillin/streptomycin (Gibco, USA) at 37°C in 5% CO2.

### Plasmids

Full-length human Sun2 cDNA (gift from Dr. Zhaocai Zhou) was cloned into a pCDH-CMV-MCS-EF1-puro vector, and oligonucleotides targeting Sun2 (shR NA 5′-GCAAGACTCAGAAGACCTCTT-3′) [13] were synthesized and inserted into pLKO.1 vectors. All recombinant plasmids were verified through DNA sequencing.

### Cell transfection and stable cell lines

The Sun2 expression or a control plasmid, the Sun2 targeting or scrambled shRNA plasmids were co-transfected with pMD.2G and PSPAX2 into HEK-293FT cells using Lipofectamine LTX (Invitrogen) according to the manufacturer's protocol. Viruses in the culture supernatants were harvested 48 h after transfection and infected cells two times at 24-h intervals. After infection, cells were selected with puromycin.

### Quantitative real-time RT–PCR

Total RNA was extracted from the fresh sample and cultured cells and quantified by qRT-PCR as described previously [10]. The primer sequences for amplification were Sun2, SAA1 and GAPDH [13, 14]. The PCR conditions were as follows: 94°C for 10min, then 40 cycles of 94°C for 30 sec, 55–58°C for 30 sec, and 72°C for 45 sec, followed by 72°C for 10 min. The quantity of each gene was normalized against GAPDH.

### Western-blot analysis

Western-blot analysis was performed according to standard protocols as described previously [11]. In brief, membranes (PVDF membranes) were incubated with antibodies against Sun2 (ab124916, Abcam, UK), α-tublin (H3663, Sigma Aldrich, USA) or SAA1 (ab201660, Abcam, USA). The intensities of light-emitting bands were checked and quantified using AmershamImager 600 (GE Healthcare,USA). α-tublin was used as an internal control.

### Cell proliferation assay

For cell proliferation assay, cells (2 × 10^3^ cells/well) were seeded in 96-well plates. Cell viability was determined using the Cell Counting Kit-8 (Sigma, China) according to manufacturer's instruction. Etomoxir (ETO) was purchased from Sigma (Sigma Aldrich, USA).

### Cell cycle and cell appotosis analysis

For cell cycle assay, The cells were harvested and fixed in 70% ethanol at 4°C for at least 24 h. Cells were subsequently resuspended in PBS, incubated with propidium iodide (PI) (Abcam, USA) in the presence of RNase A for 30 min in the dark, and finally subjected to flow cytometry analysis. For cell apoptosis analysis, cells were harvested and stained with PI and annexin V-FITC (Abcam, USA), incubated at room temperature in the dark for 15 min, and finally subjected to flow cytometry analysis.

### ATP measurements

The ATP determination kit (Beyotime Biotechnology, China) was used to evaluate the ATP measurements. The ATP concentration was determined based on comparison with a concurrent standard curve.

### The glycolytic rate and fatty acid oxidation assay

The extracellular acidification rate (ECAR) and Oxygen consumption rate (OCR) were used to evaluate the capacity of the glycolytic rate and FAO respectively by the Seahorse XF96 extracellular flux analyzer. Briefly, 2 × 10^4^cells per well were seeded overnight in XF96 well plates. For Glycolytic rate assay, One hour before XF assay, cells were equilibrated with unbuffered DMEM and maintained at 37°C for PH stabilization. Analyses were performed both at basal conditions and after injection of OLI (1 mg/ml), FCCP (1 mM) and Antimycin A (5 mM). For Fatty acid oxidation assay, the FAO assay KHB buffer supplemented with 2.5 mM glucose, 0.5 mM carnitine and 5 mM HEPES was added and adjusted to pH 7.4 in a 37°C incubator. To examine free fatty acid oxidation, BSA conjugated palmitate (Seahorse Bioscience) was added to a final concentration of 50 μM. Basal OCR of cells treated with Palmitate-BSA or BSA vehicle alone was measured.

### Tumorigenicity assay in nude mice

The cells were resuspended in a solution of Matrigel (BD, USA) and PBS (equal volume), and then injected subcutaneously into the flanks of 8-week-old male BALB/c nude mice at 3 × 10^6^ cells per site. To evaluate the tumor formation abilities of cells transfected with Sun2 plasmid or Sun2 shRNA, all mices were sacrificed in 5 weeks and the tumor were weighed.

### RNA sequencing by Illumina HiSeq

In beif, total RNA of each sample was extracted using TRIzol Reagent (Invitrogen)/RNeasy Mini Kit (Qiagen)/other kits. Total RNA of each sample was quantified and qualified by Agilent 2100 Bioanalyzer (Agilent Technologies, Palo Alto, CA, USA), and 1% agrose gel. 1 μg total RNA with RIN value above 7 was used for following library preparation. Next generation sequencing library preparations were constructed according to the manufacturer's protocol (NEBNext^®^ Ultra^™^ RNA Library Prep Kit for Illumina^®^). Sequencing was carried out using a 2 × 150 bp paired-end (PE) configuration; image analysis and base calling were conducted by the HiSeq Control Software (HCS) + OLB + GAPipeline-1.6 (Illumina) on the HiSeq instrument. The sequences were processed and analyzed by GENEWIZ INC (Suzhou, China).

### Statistical analysis

Data are expressed as mean ± SD. Differences in mRNA detection and H-score between prostate cancer and self-paired normal tissues were assessed by paired *t*-test. The chi-squared test was used to assess the correlation between Sun2 expression and patient clinicopathological characteristics. Kaplan-Meier method and log-rank tests were performed to assess the survival rate and compare differences in survival curves. Stepwise Cox proportional hazard models were used to analyze independent predictors associated with overall survival (OS). Others were analyzed using Student's *t*-test. A value of *P* < 0.05 was considered to be significant.

## Abbreviations

OS: overall survival
FAO: fat acid oxidation
FASN: fatty acid synthase
CLY: ATP citrate lyase
ACACA: acetyl-CoA carboxylase α
apoA1: apolipoprotein A1
HDL: high density lipoprotein
AMACR: α-methylacyl-CoA racemase
PET: positron emission tomography
PSA: Serum prostate-specific antigen
SAA1: Serum amyloid A1
SAA2: Serum amyloid A2
AXL: receptor tyrosine kinase
RAB1A: Ras-related protein Rab-1A
ETO: Etomoxir
NE: Nuclear envelope
ONM: Outer inner nuclear
INM: Inner nuclear membrane
PNS: perinuclear space
LINC: linker of nucleoskeleton and cytoskeleton
pT: postoperative T stage
